# Clinical use of coping in affective disorder, a critical review of the literature

**DOI:** 10.1186/1745-0179-1-20

**Published:** 2005-10-07

**Authors:** Maj Vinberg Christensen, Lars Vedel Kessing

**Affiliations:** 1 Department of Psychiatry, Rigshospitalet, University Hospital of Copenhagen, Blegdamsvej 9, DK-2100 Copenhagen, Denmark

**Keywords:** Affective Disorder, Bipolar Disorder, Depressive Disorder, Manic-depressive Illness and Coping.

## Abstract

**Background:**

The relationship between life stressors, coping and affective disorder is interesting when predicting onset of a affective disorder and relapse of mood episodes.

**Methods:**

A litteratur review of cross-sectional and longitudinal studies concerning coping and affective disorder in adults including a Medline and Embase search was conducted.

**Results:**

11 cross-sectional studies and 17 longitudinal studies concerning affective disorder and coping were found, among these, two studies include patients with bipolar disorder exclusively. Only four studies elucidate whether emotion-oriented and/or avoidance coping styles are associated with a higher risk of developing affective disorder, so this hypothesis remains unclear. Most studies shows that emotion-oriented and avoidance coping strategies are associated with relapse of depressive episodes. Conversely, problem-focused and task-oriented coping seem to be associated with a good outcome.

**Conclusion:**

There is a gap between coping theory and clinical use of coping and the clinical relevance of coping is, though promising, still unclear. In future research it is recommended to concentrate on development of a semi-structured interview combining coping style, life events and personality traits.

## Introduction

Psychosocial stressors may precipitate depression [1,2] and presumably also mania [3]. However, most persons exposed to stressful events do not develop psychiatric impairments [4]. This raises the question: why do some people experience an affective disorder in relation to a stressful life event and others do not? The answer is complex; it involves genetic loading, personality, prior experiences, parental style, social network and probably how individuals deal or cope with stress.

Coping and stress are aspects of life and coping style plays an important role in individual well-being. As formulated by H. Selye "that although we cannot avoid stress as long as we live, we can learn a great deal about how to keep its damaging side effects "distress" to a minimum" [5]. Freud's interest in defence mechanisms had a close connection to what today is named coping [6]. He believed some defence mechanisms to be healthier or less regressed than others in the same way that some coping styles seem to be associated with a healthier outcome. Synonyms for coping may be mastery, adaptation or behavioural style. Coping is defined in various ways: "any response to external life strains that serves to prevent, avoid or control emotional distress" [7], or as "constantly changing cognitive and behavioural efforts to manage specific external and/or internal demands that are appraised as taxing or exceeding the resources of the person" [8].

The failure of coping to deal adaptively with stress may lead to mental and physical illness. In general, people who rely more on approach coping tend to adapt better to life stressors and experience fewer psychological problems [9]. Approach and task-oriented coping are strategies involving problem solving, seeking information and attempts to alter the situation [10]. Conversely, avoidance and emotion-oriented coping strategies seem to be associated with psychological distress [11]. Avoidance coping describes activities aimed at avoiding the stressful situation and involves denial, wishful thinking and withdrawal [12]. Emotion-oriented coping describes emotional reactions that are self-orientated in order to reduce stress. These reactions involve emotional responses (individuals blaming themselves for being too emotional, becoming angry-, or tense) [13] and ruminative responses defined as: behaviours and thoughts that focus attention on depressive symptoms and on the implication of these symptoms (individuals thinking how tired they feel and why they get depressed, why others do not) [10].

Many studies of coping examine the relationship between coping and somatic illness. Research on the relation of coping and depressive disorder has attracted attention, but in clinical psychiatry there is a gap between clinical practice and research [14,15]. With changing treatment approaches for depressive and bipolar disorders such as interpersonal psychotherapy and cognitive-behavioural therapy coping may be important as a target for treatment. Therefore clinical studies involving coping and depressive and bipolar disorders in adults are reviewed in order to investigate two hypotheses; Are avoidance and emotion-oriented coping strategies associated with an increased risk of developing an affective disorder and with a higher risk of relapse of mood episodes?

## Methods

Electronic searching in Embase and Medline using the combination of the following terms: Mood Disorders or Bipolar Disorder or Depression (MeSH-terms) Mania or Manic-depressive Illness (Text-words) and Coping (Text-word): 5864 hits Medline, 3016 hits Embase. Studies of coping and affective disorder were selected by reading headings and abstracts. The following studies were excluded; coping and somatic illness, coping in children and adolescents and studies of coping concerning individuals as employees, parents etc. Medline Index starts in 1968. The search was ended at March 2005.

## Results

As seen from the Tables, 11 cross-sectional studies and 17 follow-up studies were identified. The studies are presented in three tables according to design and in historical order. Five studies investigated the same sample [16,35,36,40,43].

### Cross-sectional studies

The 11 cross-sectional studies are presented in Table 1. The cross-sectional studies showed; **Firstly**, a relation between emotion-oriented and avoidance coping strategies and the prevalence and symptom severity of depression. **Secondly**, the cross sectional studies cannot show whether these strategies were associated with first episode or with relapse of a depressive or manic episode.

**Table 1:** Cross-Sectional studies of the relationship between mood disorder and coping style.

Study Controlled = CON	Patients No.	Sex Men %	Mean Age	Measures	Results
Billings [16] 1984 CON	424 outpatients	55	41 yr	HDLF RDC	Coping responses directed toward problem solving and affect regulation were associated with less dysfunction. Emotional discharge responses were linked to greater dysfunction.
Rosenberg [17] 1987 CON	24 inpatients. 36 controls	48	36 yr	BDI CR	Depressed patients more often reported avoidance strategies to cope and non-depressed utilize active coping techniques
McNaughton [18] 1992 CON	27 inpatients	100	42 yr	RDC, HRDS LEDS, WCC	The depressed group used more emotion-focused coping than control persons.
Turner [19] 1992	26 outpatients	31	39 yr	BDI MCI	Significantly positive correlation between high depression scores and emotion oriented coping. Significantly negative correlation between high depression scores and task oriented coping.
Lam [20] 1997	40 bipolar patients	43	44 yr	MRC, IQ MA, BDI CPI	Patients' level of social functioning was related to their level of insight and how well they coped with prodromes of mania and whether they could detect prodromes of depression.
Dekker [21] 1999 *	248 outpatients major depression.	42	Ca 35 yr	HDRS SCL-90 QLDS UCL	The more depressed patients used passive reaction pattern and avoidance significantly more often than less depressive patients. Passive reaction was the most consistent predictor of high depression score.
Schouws [22] 2001	211 outpatients with major depression.	42	34 yr	DSM-III HDRS, UCL SCL-90, QLDS	Active approach and seeking social support associated with a higher quality of life. No gender differences in coping. Avoidance coping was related to higher severity of depression.
Vossler [23] 2001 CON	41 in and out patients, 41 control persons	53	42 yr	SCID F-SOZU FKV-LIS	Depressive patients reported more social stress and used ineffective coping strategies and wishful thinking more often than the control persons.
Ravindran [24] 2002 CON	229 dysthymic, major depressive. 44 controls	39	41 yr	HAM-D, BDI MADRS, CGI, CGIS, DHUS	Among depressive patients the severity of illness was associated with emotion focused coping, whereas control-persons favoured the use of cognitive strategies.
Lam [25] 2003	109 outpatients unipolar	45	44 yr	SCID, HRSD, RSQ IIP, SPC	Rumination was associated with higher levels of depression. Distraction was associated with lower level of depression.
McWilliams [26] 2003	298 outpatients major depression.	48	43 yr	CISS NEO-FFI BDI, STAI-S	Task-oriented and social coping were associated negatively and emotion-oriented coping was associated positively with high scores on depression and anxiety.

* Used a comparison group from another study.

BDI Beck Depression Inventory. Depression Scale. CGIS Clinical Global Impressions Scale. CIDI Composite International Diagnostic Interview-Short Form.

CISS Coping Inventory of Stressful Situations. CPI Coping with Prodromes Interview. CR Coping Response. CSS Coping Strategies Scale.

DSM-IIII Diagnostic and Statistical Questionnaire Manual of Mental Disorders fourth edition. DCS 8 8 Different Coping Strategies. DHUS Daily Hassles and Uplifts Scales.

HPS Hypomanic Personality Scale. HAM-A Hamilton Anxiety Scale. HDLF The Health and Daily Living Form. HRSD Hamilton Rating Scale for Depression.

IIP Inventory of Interpersonal Problems. IQ The Insight Questionnaire. LE Life Events over the past year. LEDS Life Events and Difficulties Schedule.

MADRS Montgomery Asberg Depression Rating Scale. MAS The Mania Scale. MCI The Multidimensional Coping Inventory. MRC Social Performance Schedule.

NEO-FFI NEO Five Factor Inventory. QLDS Quality of Life Depression Scale. RCD Research Diagnostic Criteria. RL Recent Life events and Chronic Stress.

RSQ Nolen-Hoeksema Response Styles Questionnaire. SCID Structured Clinical Interview for DSM-IV SCL-90 Symptom Check List of 90 items.

SPS Social Performance Scale. STAI-S. The State Anxiety Subscale of the Spielberger State-Trait Anxiety Inventory. UCL Utrecht Coping List. UCLA Loneliness Scale. WCC The Ways of Coping Checklist.

### Short-term studies

The seven short-term studies are presented in table 2. Short-term studies are here defined as studies with a three- to six-month follow-up period. The short-term studies indicated: **firstly**, in four of the seven studies that emotion-oriented and ruminative coping style were state dependent [27,29,31,32], **secondly**, in four studies these coping styles were related to the severity of the depressive symptoms [27,29-31], **thirdly**, three studies showed that recovery from depression were associated with changes in coping style [27,29,31], accordingly, recovered patients relied less on inappropriate emotion-focused coping strategies and **finally**, two studies showed that emotion-oriented coping predicted the presence of depression at follow-up [28,30]. Conversely one study showed that ruminative coping might be associated with a good outcome in depressed patients [33].

**Table 2:** Short-term (3–6 months] studies of the relation between coping and mood disorder.

Study Controlled = CON	Follow-up month	Patients No.	Sex % Men	Mean age	Measures	Results
Parker [27] 1982, CON	4	95 from a non-clinical group	33	38 yr	BCG EF	Disturbances in antidepressive behaviours were more likely to be a consequence of rather than an antecedent of depression
Parker [28] 1986	4	43 depressed 66 volunteers	17 39	32 yr 42 yr	BDI, PSE CQ, ZDS	Higher self-consolation scores predicted less improvement. Those who scored low on three subscales self-consolation, distraction and socialization had better improvement
Schussler [29], 1992	2	40 depressed patients		45 yr	DS ADV-L	Found difficulty in distinguish between symptoms of depression and certain coping behaviours [e.g. withdrawal]
Ravindran [30] 1995 CON	6	17 dysthymia 17 depressive and 18 controls.	49	38 yr	MADRS, CSS HAM-A, CGIS DHUS, UCLA	Recovery from depression was associated with change in coping style, so that patients relied less on inappropriate emotion-focused coping strategies.
Kuehner [31] 1999	4	52 unipolar	42	44 yr	SCAN, PSE-10 IDD, RSQ	A diagnosis of depression was associated with rumination. Baseline rumination predicted follow-up levels of depression.
Uehara [32] 2002	4	36 depressed, 13 anxiety	39	39 yr	CISS, HRSD SAS, DSM-III	Task-oriented coping was influenced by depression. Emotion-oriented coping was influenced by anxiety. State and treatment phase affected coping measurement
Yamada [33] 2003	6	105 depressed patients	42	44 yr	COALA GAS HRDS	Patients with a good outcome at 6 months used significantly more rumination while patients with a poor outcome used significantly more dangerous activity

ADV-L Die Liste Antidepressiver Verhaltensweisen von Hautzinger. BDI Beck Depression Inventory. BCG Behavioural Change Questionnaire. CISS Coping Inventory for Stressful Situations.

COALA Comprehensive Assessment List for Affective Disorders. CQ Coping Questionnaire. CSS Coping Strategies Scale. DHUS Daily Hassles and Uplifts Scales.

DS Depressionsscale von v. Zerssen. DSM-IIII Diagnostic and Statistical Questionnaire Manuel of Mental Disorders fourth edition. EF Effectiveness Scale.

EHEI Early Home Enviroment Interview. GAS Global Assessment Scale. HAM-A Hamilton Anxiety Scale. HRSD Hamilton Rating Scale for Depression. IDD Inventory to Diagnose Depression.

LIFE Longitudinal Interval Follow-up Evaluation. MADRS Montgomery Asberg Depression Rating Scale. RSQ Response Style Questionnaire. SAS Self-rating Anxiety Scale.

SCAN PSE 10 Schedules for clinical assessment in Neuropsychiatry with the Present State Examination, 10 th edition. SCID Structured Clinical Interview for DSM-III. ZDS Zung Depression Scale.

### Long-term studies

The 10 long-term studies are presented in table 3. Long-term studies are here defined as studies with a follow-up period longer than six months. The long-term studies indicated: **firstly**, that adaptive coping strategies predicted increased remission and decreased risk of relapse in half of the studies [35,37,39,41,42]. One study concluded that depressed persons did not differ in amount of problem-focused coping compared to control persons [34]; another study did not find that problem-focused coping was a predictor of post-treatment symptoms [38]. **Secondly**, the use of passive and ruminative coping strategies and coping by avoiding other people was found to be related to a higher risk of relapse and of greater severity of depressive symptoms at follow-up in six of the ten studies [34,35,37,39,40,42]. **Thirdly**, one study showed that a high level of stressors was connected with higher depression scores at follow-up [37]. **Finally**, the study on bipolar disorder showed that patients that used stimulating coping strategies more often had a manic episode [42]

**Table 3:** Long-term (over 6 months] studies of the relation between coping and mood disorder.

Study Controlled = CON	Follow up year	Patients No.	Sex Men %	Mean age	Measures	Results
Coyne [34] 1981, CON	1	15 depressed and 72 controls	52	55 yr	HSCL, WCL 4 weeks intervals	The coping of depressed persons was characterized by seeking emotional support and by wishful thinking.
Billings [35, 36] 1985, CON	1	380 unipolar	57	40 yr	HDL FES RDC	Patients at follow-up used significantly more affect regulation and less reliance on information seeking and emotional discharge, latter coping styles were associated with poorer outcome
Swindle [37] 1989, CON	4	352 unipolar	44	44 yr	HDL, FES RDC	Problem solving related to less depression and greater self-esteem. Emotional discharge associated with depression.
Hoffart [38] 1993	1	21 depressed 17 depr/phobia, 23 agoraphobia.	34	41 yr	SCID, WICCA BDI, ACS CPRS	Seeking social support may be a trait dependent coping style. Problem focused coping and wishful thinking appeared as a state phenomena.
Sherbourne [39] 1995	2	604 depressed	26	46 yr	DSM-III SF-36, COD	Better clinical course of depression was associated with more active and less avoidant coping styles
Moos [40] 1999, CON	10	313 unipolar	40	48 yr	DSSI, HDL RDC	Patients were at risk for a chronic course if they coped with stressors by avoiding being with people.
Oldehinkel [41] 2000	3 1/2	86 from primary care	31	37 yr	PSE, DSM, UCL LEDS, SRS, ABV	Predictors that expedited remission were high self-esteem and a tension reducing coping style.
Lam [42] 2001	1 1/2	40 bipolar	43	44 yr	MAS, SCID CPSI	More who used stimulating coping strategies had a manic relapse. More who used passive coping strategies had a depressive relapse.
Holahan [43] 2003, CON	10	313 unipolar	40	48 yr	HDL, RDC, DTC DSSI, DP	Patient who more often drank to cope at baseline had a stronger association to depressive symptoms and drinking problems.
Szadoczky [44] 2004	2	117 unipolar	25	44 yr	HRSD, MMPI, WCC, SAS, LEQ	No significant difference between the group of remitters and the group of non-remitters in problem-solving coping and emotion-focused coping.

ABV Amsterdams Biografische Vragenlijst. ACS Agoraphobic Cognition scale. BAI Beck Anxiety Inventory. BDI Beck Depression Inventory. COD Course of Depression.

CPRS Comprehensive Psychopathology Rating Scale. CPSI Coping with Prodomal Symptoms Interview. DIS Diagnostic Interview Schedule.

DSM-III & IV Diagnostic and Statistical Manual of Mental Disorders [third and fourth edition]. DP Drinking Problems. DTC Drinking to Cope. DSSI Depressive Symptoms Severity Index.

FES Family Environment Scale. GAS Global Assessment Scale. GDS Geriatric Depression Scale. HSCL Hopkins symptom Checklist. HDL Health and Daily Living Form.

HRSD Hamilton Rating Scale for Depression. MAS Mania Scale. LEQ Life Event Quistionnaire. PB Passive Behaviours. MMPI Minnesota Mulriphasic Inventory. PSE Present State Examination.

RDC Research Diagnostic Criteria. SADS-L Schedule for Affective Disorders and Schizophrenia-Lifetime. SAS Social Adjustment Scale. SCI Stress Coping Inventory.

SCID Structured Clinical Interview for DSM-III. SF-36 a 36-item Self-report health-related quality of life measure. Social Support and Rejection Scale. UCL Utrecht Coping List.

WCC The Ways of Coping Checklist. WWC L The revised Ways of Coping Checklist.

### Results summary

Regarding the two hypotheses none of the studies were capable of answering whether certain coping strategies are predictive of affective disorder. Most studies showed that in depressive disorder avoidance and emotion-oriented coping strategies seem to be related to recurrence/relapse of depressive episodes. These coping strategies might also be associated with an increased time to recovery. Only a few studies on bipolar disorder were identified, limiting the findings to depressive disorder mainly. In the cross-sectional and short-term studies it is difficult to make a clear distinction between coping processes and symptoms, and avoidance and emotion-oriented coping styles seemed to be state related, so the results of these studies are not able to answer our hypothesis.

## Discussion

### Study-design

Regarding the trait or state effect, the use of cross-sectional designs and designs with a short follow-up period can be problematically because of the possibility of measuring residual depressive symptoms. The coping style of an individual may change during a depresive or manic episode, as illustrated in the short-term studies. Possibly, the emotion-oriented coping style found especially in the cross-sectional studies could be explained by a state-dependent consequence of a depressed mood. One way to determine whether the anomalies are caused by the depressive or manic episode (state model) is to include a control group. Among the 28 studies, 12 included a control group. In the follow-up studies, the lack of a control group makes it difficult to distinguish whether the changes that occurred reflect normalization of coping over time or whether the changes reflect changes in affective symptoms. The best impression of the relation between coping and state comes from the follow-up studies that used repeated measures of affective symptoms, life events and coping behaviour and include a control group [34-37,40,43]. The studies with a long follow-up time [37,40,41,43] illustrate the time-relation between coping and affective episodes: these studies support the hypothesis that emotion-oriented and avoidance coping strategies seem to be associated with increased risk of relapse. So, the controlled follow-up studies provided the most valid study design to comply with methodological problems.

### Comparing results

It is difficult to compare studies involving heterogeneous samples and it is likely that selection bias influenced the results unpredictable. Most studies used clinical samples of unipolar patients. A general limitation is the lack of attention to confounding variables such as the duration and severity of the mood episodes and the number of previous episodes. Some studies investigated these parameters [21,23,28,30,39-41] and these results did not deviate substantially from those of other studies. The use of passive coping strategies predicted relapse of depression and stimulating coping strategies increased the relapse risk of mania but from the limited number of studies concerning bipolar disorder [20,42] it is not possible to elucidate the relation between coping and bipolar disorder. The little attention to research into coping mechanisms and mania was already stressed in a case report in 1990 [45] and latest by the authors in a research report from 1999 where the development and validation of the coping inventory for prodomes of mania was described [46].

Participants were registered differently according to comorbid psychopathology. In one study the most common comorbid disorders were panic disorder, dysthymia and social phobia [26]. One study concentrated on comorbid agoraphobia [39] and one on comorbid personality disorder [44]. In some studies patients with comorbid psychopathology were excluded [16,21,22,28,29]. Although the relation of coping processes and risk for substance abuse is a defined area of coping research [47], only two studies [39,43] adjusted for the possible effect of comorbid substance use as a part of a coping strategy. This may have affected the results so that some inappropriate coping strategies are not identified.

### Coping measurements

As seen from the tables, the studies measured coping differently, which makes it difficult to compare across studies. The coping measures were based on self-reports and a variety of questionnaires were used without any golden standard. It is important to develop reliable and valid tools that assess how people cope with stressful situations and negative events. Therefore developing structured and easily scorable scales started in the 1960s [48] and during the 1980 and 1990s, – much research was conducted on self-reported measures of coping. Facing particularly stressful events, individuals are asked about the kind of coping behaviour(s) in which they have engaged [49]. This research used widely divergent strategies, techniques and measures; additionally some of the scales possess a variety of psychometrically drawbacks [13]. When examining the coping questionnaires, it is clear that substance use as a coping behaviour has virtually been ignored [13,27,50,51]. The two studies concerning abuse showed that depressive symptoms were significantly associated with more drinking problems [43] and that improvement in functioning and well-being was associated with less alcohol consumption [39].

As discussed, in a critical review concerning the gap between coping theory and clinical intervention research [52], when using a coping checklist and thereby reducing coping to a summary score, a lot of information will be lost. The questions seem to be to general for the results to be valid and crucial aspects of timing, sequencing and appropriateness may be lost.

### Coping and life events

There is a substantial and partially causal link between life-events and depression [53] and the occurrence of major life events signals a period of increased risk of developing a depressive episode [54]; several studies measured life events [17,18,21,23,24,26,27,29,34-37,39,43,44]. A general problem in measuring coping in connection with life events is the time relation, as participants should recall a stressful situation and then reconstruct how they dealt with it. Firstly, there is recall bias and secondly, coping has a temporal aspect: an individual can cope before a stressful event occurs, while it is happening and afterwards [55]. The issue of timing is complex as there presumably may be an interaction between individual vulnerability, coping, life events and the risk of developing depression. Thus it is difficult to measure the coping process given the typical brief period between a severe life event and depression. Capturing the coping process represents a challenge [56] and it may require a more frequent monitoring of coping than is usually done in studies.

All coping measures are based on self-reports and participants are often asked in connection with hypothetical stressors e.g. the Coping Inventory for Stressful Situations [13] and it is difficult to know what kind of stressors were thought of. Many scales for coping are developed to assess overall coping strategies but it reasonable that human cope differently according to the nature of the event. Linking coping measures to recent actual stressors and attempting objective assessment of the stressful life event to avoid circularity is of main importance. The procedure of semi-structured interviews is time- and labour consuming and might be a reason for the consistent use of self-reports. The lacking use of coping in clinical assessment and treatment may be due to the rather confusing divergent coping scales. It can be rather meaningless to detect coping not knowing what kind of stressor the coping process relates to and the influence of personality traits cannot be ignored [56]. For advancing research and clinical focus on coping there is a need of developing an integrated semi-structured interview detecting personality traits, life events and coping together. Beside, a specific development of coping schedules capable of identifying prodomal symptoms of bipolar episodes as described by Wong et al. [46] would be of clinical use.

### Coping and personality

Coping is closely related to personality and personality can affect coping measures and in coping as well as in personality research there is a distinction between trait and state variables. In the concept of coping responses [57], coping is seen as a response to specific stressful situations rather than a stable feature of personality. Another approach emphasizes the method of coping; whether a response entails primal cognitive or behavioural efforts [58]. One study [26] showed that less-adaptive coping strategies (emotion-oriented coping) were associated with neuroticism and depression, whereas the reverse association was found regarding adaptive coping strategies (task-orientated coping). These findings are replicated in studies of the general population [59,60]. Finally, a study [61] from Japan found that task-oriented and avoidance coping were related to extraversion and that emotion-oriented coping was related to neuroticism. Personality traits are important in the assessment of coping and it is advisable to measure these traits when dealing with coping measures; however, only three studies have done so [26,41,44].

### Coping and Gender

In most of the present studies more women were included reflecting the gender differences in the prevalence of depressive disorder. In some studies gender differences were found and the general tendency was that men tend to distract themselves using active coping strategies, whereas women use strategies involving expressing emotion [16,21,25]. Other studies found no gender differences [22,30,33]; however, most studies did not take gender into consideration in the analyses. According to the hypothesis of Nolen-Hoeksema [62] the increased vulnerability of women to developing depression is related to gender differences in coping; men's response to their dysphoria is more behavioural and dampens their depressive episodes, whereas women's response to their dysphoria is more ruminative and amplifies them. Accordable to a review [63] of gender differences in depression, it is possible that men tend to distract themselves from their mood by engaging in physical or instrumental activities, whereas women are less active and ruminate over the possible causes and implications of their depression. These hypotheses are compatible with findings from other studies [64-66] the latter study involving data covering representative population samples from six European countries. Conversely, an older prospective one-year study [67] of 100 healthy community-residing men and women, in which participants were interviewed seven times at four-week intervals, found no gender differences in emotion-focused coping. Another study [68] of couples that recently had experienced at least one threatening life event that was potentially depressonigenic for both showed that women had a greater risk than men of depressive episodes following the life event. The greater risk was restricted to episodes that followed events involving children, housing or reproductive problems. Women's greater risk was only present among those couples for whom there were clear gender differences in associated roles.

### Coping and age

Age differs between the studies but most studies had a broader age range. How age influences the stress and coping process is not clear: The Normative Aging Study [69], a longitudinal study examined stress, appraisal and coping in three groups' middle-aged, young-old and old-old men. The study followed 2280 men for more than 30 years. A significant overall effect of age on coping strategies: instrumental action, cognitive reframing, social support and interpersonal hostile strategies were found and all coping strategies showed linear decrease with age. The relation between age and coping is complex and there is no clear answer as to whether persons cope better or worse as they age. In a recent study, the association between life events and onset of depression and mania was not found to change throughout life [70]

### Coping and medical adherence

The episodic course of affective disorders presents a challenge to the clinician and the patient, in making and fulfilling a treatment plan. Effective treatment depends on medication adherence also when treating medical patients with comorbid depression. When treating affective disorder, doctors and patients have to deal with different problems: lack of insight, symptom-free intervals, residual affective symptoms and poor social support, which can complicate long-term treatment [71]. Identification of these factors may add to increase medication adherence as seen in a study [72] that investigated medication adherence in 32 patients with bipolar I disorder. Consistent with the hypothesis that acceptance coping would be positively associated and denial coping negatively associated with adherence to mood-stabilizing medication, it was found that low levels of acceptance and a high level of denial undermined medication adherence.

### Clinical implications

Coping strategies could be a target for selective prevention targeting subgroups of the population whose risk of developing an affective disorder is higher than average. Further, a way to ameliorate the course in affective disorder is to help patients to identify prodomal symptoms and individual maladaptive coping strategies and try to change these, e.g. problem training as a treatment for depression [46,73,74] or intervention designed to improve coping. More recent literature supports the utility of individual cognitive behavioural and psycho-educational approaches, particular in enhancing medication adherence, so medication and psychotherapy are not only compatible but also synergistic [75] and a strong focus in psychological treatment involves structured attempts to teach patients new coping skills.

## Conclusion

The gap between coping theory and clinical use of coping remains and the clinical relevance of coping is, though promising, still unclear. It is difficult to make a clear distinction between coping processes and symptoms. The complex interaction between life stressors, coping, personality and affective disorders need to be better understood before coping behaviour and changes in coping strategies can be included more systematically in patient treatment. Primary, there is a need to develop new valid and reliable measures combining assessment of coping, life events and personality in a semi-structured interview. Long-term high-risk studies capable of detecting coping before and after an onset of an mood disorder would provide new information of the relation between coping strategies and affective disorder.
